# Relationship Between Dietary Patterns and Dental Health in Type I Diabetic Children Compared With Healthy Controls

**DOI:** 10.5812/ircmj.9684

**Published:** 2014-01-05

**Authors:** Leila Bassir, Reza Amani, Mashalla Khaneh Masjedi, Fatemeh Ahangarpor

**Affiliations:** 1 Departmant of Pedodontics, Jundishapur University of Medical Sciences, Ahvaz, IR Iran; 2 Departemant of Nutrition, Jundishapur University of Medical Sciences, Ahvaz, IR Iran; 3 Departemant of Orthodontics, Jundishapur University of Medical Sciences, Ahvaz IR Iran

**Keywords:** Diabetes Mellitus, Dental Caries, Diet

## Abstract

**Background:**

Dietary habits are established in childhood and will persist until adulthood, being one of the human health pillars. Many diseases of humans have roots in the individuals’ diet, of which dental caries are one of the common infectious diseases. Diabetes Mellitus is also considered as the most common metabolic disorder in children.

**Objectives:**

The purpose of this study was to compare the dietary patterns of children with type I Diabetes Mellitus with that of non-diabetic children, in relation to dental caries.

**Materials/Patients and Methods:**

In this study, 31 patients (13 boys and 18 girls, mean age of 11 ± 5.4 years) with type I Diabetes Mellitus referred to the Diabetes Mellitus Center and university hospitals were selected. Controls were 31 healthy students matched for age and sex. The study was based on the data obtained from the questionnaire containing information about dietary patterns and oral hygiene habits, social class and decayed/missing/filled teeth (DMFT) index. Dietary patterns were assessed using a food frequency questionnaire developed on the basis of caries preventing or inducing foods and then scored. Data were analyzed by using the t-test and McNamara’s test.

**Results:**

Diabetic children had less frequent cariogenic snacks than their controls. The mean diet scores for diabetic and healthy subjects were 7.65 ± 3.27 and 11.9 ± 2.03 (P < 0.05), respectively. There was no significant difference in DMFT between the diabetics and controls (3.71 ± 2.48 vs. 4.35 ± 2.74, respectively). There were also no differences in frequency of tooth brushing and use of mouth washes. However, more diabetics reported that they have never used dental floss compared to controls (42.2% vs. 71%, P < 0.05). Having cheese with bread as snack was more prevalent in diabetics (P < 0.05).There was a positive correlation between DMFT and dietary scores (r = 0.3, P < 0.05).

**Conclusions:**

Controls scored higher in their dietary habits and dental flossing but lower in tooth brushing and mouth washing. More diabetics tend to have snacks like cheese and bread, which is a caries-preventing habit.

## 1. Background

Dietary habits are established in childhood and will persist until adulthood, being one of the human health pillars. Many diseases of humans have roots in the individuals’ diet, of which dental caries are one of the common infectious diseases. Diabetes Mellitus is also considered as the most common metabolic disorder in children. Several researches have been conducted to explore the relationship between these two diseases that lead to different results.

Canepari et al. conducted a study on counting Streptococcus mutants and Lactobacilli from the saliva and analyzed their relation with dental caries in children with type I Diabetes Mellitus. They showed that if diabetic and healthy children use the same diet, the dental caries would be more prevalent in diabetic children (1). Ciglar et al. in a study on 84 diabetic patients (60 patients with type I and 24 patients with type II Diabetes Mellitus), showed that daily intake of carbohydrate and simple sugars was much lower in diabetic patients than non-diabetic, and dental caries location was found to be more prevalent on the buccal and labial cervical area in diabetic patients (2).

Siudikiene et al. evaluated the relationship between diet and dental caries in children with type I Diabetes Mellitus and found that the increase in age and frequent consumption of soft drinks and snacks can influence dental caries development in children (3). Samimi and colleagues investigated the prevalence of dental caries and DMFT values among 151 diabetic children (ages 6–12 years) in Iran, and observed that an earlier disease onset is associated with the greater mean of DMFT (4). Siudikiene et al. suggested that dental plaque and Diabetes Mellitus induced changes in albumin concentration and salivary glucose, induced dental caries among diabetics (5). Alavi et al. conducted a study to evaluate the prevalence of dental caries in 50 type I diabetic children aged 5–18 years and found that the dental caries rate was higher in diabetic children compared with that of healthy individuals (6). At present, it is unclear whether Diabetes Mellitus may increase the risk of dental caries or diabetic patients' dietary patterns may be the cause.

## 2. Objectives

The purpose of the present study was to compare the diet of children with type I Diabetes Mellitus with that of non-diabetic children, with regard to its relationship with dental caries.

## 3. Materials and Methods

In this study, 31 patients (13 boys and 18 girls), with mean age of 11 ± 5 years within the range of 7–17 years, diagnosed with type I Diabetes Mellitus referred to the Diabetes Mellitus Center and university hospitals, were randomly selected. This group was age and sex matched to 31 healthy students from the same area, as the control group. The diabetics had no other systemic disease and their blood glucose were check and record and controls were students randomly selected from the local school, also with no systemic disease. There was no tooth structural anomaly in the two groups.

The study was approved by Ahvaz Jundishapur University of Medical Sciences Ethics Committees and all subjects’ parents gave their informed consent for participation. Subjects were selected through random sampling in both groups. Each patient underwent a dental caries assessment for the DMFT index according to the WHO recommendation form (7). The questionnaire used included personal information regarding oral hygiene status (such as use of dental brush, dental floss, and mouth wash) and cariogenic foods, obtained through a food frequency questionnaire (8). This questionnaire consisted of 11 dental caries-inducing and four dental caries-preventing foods, in which food items were scored 1–4 based on the frequency of use per day or week, as follows: score 4 – frequent use in a day; score 3 – once a day; score 2 – three–five times a week; score 1 – less than three times a week . Positive and negative scores were given to caries-inducing and caries-preventing foods, respectively (9). Statistical analyses: Data were analyzed using SPSS version 16.0 software (IBM Corp., NY, USA). In order to compare the means of independent variables and the ratios, the t-test and McNamara’s test were used, respectively. The P-values less than 0.05 were considered as significant.

## 4. Results

In diabetic group, the use of tooth brush, dental floss and mouth wash were 77.4 % (24), 42.2 % (14) and 38.7 % (12), respectively. These variables in controls were 80.6 % (25), 71 % (22), 35.2 % (11), respectively. Only the use of dental floss was significantly different between the two groups (P < 0.05). The mean score for using caries-causing food was significantly higher in the control group compared with the diabetics (11.94 ± 2.03 vs. 7.65 ± 3.27 (P < 0.05), respectively,). The mean DMFT for the diabetic and healthy groups were 3.71 ± 2.48 and 2.74 ± 4.35, respectively, which were not significantly different (Table 1). 

**Table 1.** Age and DMFT Components of Diabetic and the Control Group Variables

Variables	Diabetic Group, Mean ± SD, n = 31	Healthy Group, Mean ± SD, n = 31	P Value
**Age, y**	11.3 ± 2.5	11.2 ± 2.4	0.9
**Decayed**	2.41 ± 1.92	2.87 ± 1.12	0.38
**Missing**	0.32 ± 0.54	0.32 ± 0.54	1.0
**Filled **	0.96 ± 1.1	1.2 ± 1.13	0.25
**DMFT **	3.71 ± 2.48	4.35 ± 2.74	0.33

**Figure 1.**
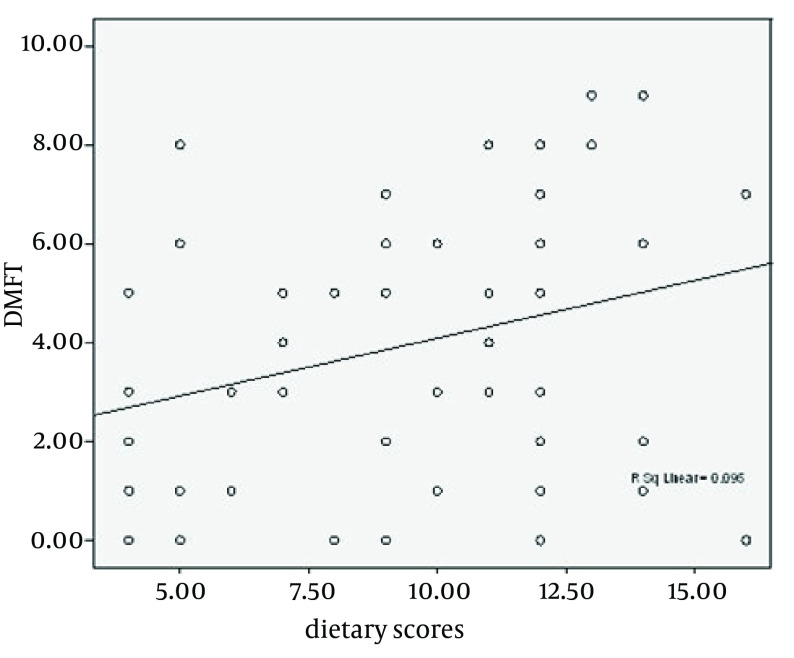
Linear Correlation Between DMFT and Dietary Scores (r = 0.3, P < 0.05)

Furthermore, the mean of DMFT showed no significant difference between diabetic and healthy groups in relation to gender (Table 2). The comparison of dental caries inducing and preventing foods for both diabetics and controls revealed no significant difference between the groups, except for consumption of cheese and bread as snack (P < 0.05) (Table 3), which was higher for diabetics. More controls had biscuits as snacks (P = 0.053) compared with diabetics. According to (Figure 1), there is a significant correlation between DMFT and dietary scores (r = 0.3, P < 0.05). The higher the scores are, the higher the dental caries inducing foods consumption is.

**Table 2.** Comparison of Dietary Patterns and DMF Score Between Diabetic and Healthy Groups in Relation to Gender

Variables	Diabetic Group, Mean ± SD		Healthy Group, Mean ± SD		P Value
	Male	Female	Male	Female	
**DMFT**	3.54 ± 2.78	3.83 ± 2.30	3.54 ± 2.78	4.94 ± 2.66	0.2
**Dietary score**	7.92 ± 3.22	7.44 ± 3.38	11.92 ± 2.53	11.94 ± 1.66	0.8

**Table 3.** Comparison of Caries-Inducing and Caries-Preventing Foods Between Diabetic And The Control Groups

Caries-Inducing Items	Diabetic No. (%)	Healthy No. (%)	P Value
**Biscuits**	13 (42%)	16 (51%)	0.053
**Chocolate **	1 (3%)	8 (26%)	0.636
**Ice cream**	0 (0%)	6 (19%)	0.664
**Soft drink**	0 (0%)	6 (19%)	0.664
**Sweetened hot tea**	11 (35%)	15 (46%)	0.106
**Dry fruits**	0 (0%)	2 (7%)	0.206
**Sweetened hot milk**	7 (22%)	2 (7%)	0.324
**Candies**	0 (0%)	2 (7%)	0.206
**Confectionery cookies and cakes**	1 (3%)	0 (0%)	0.200
**Canned fruits/ jams**	0 (0%)	0 (0%)	0
**Sugary gum**	1 (3%)	6 (19%)	0.402
**Caries-protecting items**			
**Nuts (Walnut and almond)**	0 (0%)	0 (0%)	0
**Cheese and bread**	13 (45%)	11 (35%)	0.027
**Apple and cucumber**	10 (31%)	7 (22%)	0.072
**Sugar-free gums**	7 (22%)	0 (0%)	0.789

## 5. Discussion

The present study showed that the mean DMFT scores are not different between diabetic and healthy children. However, more diabetic children consumed cheese and bread, as a dental caries-preventing snack, in comparison with the healthy subjects (P < 0.05). Furthermore, fewer diabetics tended to have biscuits as a snack, which is a caries-inducing food. Both habits could be regarded as dietary strategies in diabetics, which are emphasized by dentists and dietitians. In a study conducted by DoAmaral et al. on 64 non-diabetic and 30 diabetic subjects aged 17–28 years, the authors suggested that, despite having a higher frequency of meals, less tooth brushing and dental floss use, type I diabetic subjects were less prone to dental caries than non-diabetics (10). On the other hand, Wenger had shown that the prevalence of dental caries in diabetic patients is higher than in non-diabetics (11).

Tonovuo et al. showed that the prevalence of dental caries in diabetics was as high as in non-diabetics, but the past dental caries experience was remarkably lower in diabetics in which that control of diet had started at a very early age. Furthermore, they claimed that adult diabetic patients are as susceptible to dental caries as their matched healthy controls, probably due to the leakage of glucose from blood into the oral cavity (12). The result of this study was fairly close to the present study. Twetman et al. studied the risk assessment of 64 young type I Diabetes Mellitus mellitus patients (8–16 years) and suggested that a dental caries risk assessment at the diagnosis of Diabetes Mellitus mellitus in children may be a good indicator of overall health care (13). Piatelli et al. conducted an epidemiological study on 26 type I diabetics and 24 healthy subjects with similar age and sex, and observed a higher incidence of dental caries in diabetic patients than in the healthy subjects, probably due to the rich carbohydrate diet (14).

Our study showed that higher dietary scores are correlated to higher DMFT, indicating that more dental caries-causing foods intake resulted in lower dental health status (Figure 1). Albrecht and colleagues showed that a diet without sucrose cannot reduce dental caries in diabetic patients (15). In the Städtler and colleagues study, despite the patients’ good adherence to the diet, the rate of dental caries in diabetic patients was not lower than in healthy subjects (16, 17). In the studies that reported the higher rate of dental caries in diabetic patients than in healthy subjects, the main causes were decreased salivation, increased glucose concentration in saliva and, in some studies, problems of oral hygiene (6). Tagelsir et al. investigated the dental caries index in 52 children and adolescents, 3–16 years of age with type I Diabetes Mellitus and reported considerably high levels of dental decay among the diabetic children (18). In some other studies, a lower incidence of dental caries in diabetic patients compared with healthy individuals has been reported (19, 20).

Städtler et al. stated that this difference is secondary to the low cooperation of patients in treatment procedure (16, 17). In most studies, the differences of social classes between diabetic and healthy children have not been observed (3, 6), but the differences between the results in terms of oral hygiene and tooth brushing have been shown. Johns has mentioned that, despite the diabetic patients have more attention to their oral health and hygiene status, their higher risk of dental caries associated with Diabetes Mellitus, causes a more frequent occurrence of caries in these individuals than in healthy people(19). However, in other studies, the attention to oral health and hygiene status has been reported to be lower in diabetic patients (4), and this difference was not statistically significant (2, 3, 6). In the present study, significant differences in the use of mouthwash solutions and toothbrushes were not observed, but the use of dental floss amongst Diabetes Mellitus children was significantly lower than in the control group.

The DMFT in our study in both diabetic and healthy children was above the standard level reported by WHO (21), which revealed the need for more attention by health services and more effective focus on oral health in the area. There was no significant correlation between dental caries and blood glucose values, as well as the duration of Diabetes Mellitus in our cases. In agreement with this finding, two other studies showed no relation of the DMFT index with the disease (3, 13), while in another study reported a negative correlation between Diabetes Mellitus onset and DMFT (4). It is assumed that the low number of subjects could explain some of the non-significant results obtained in this study, and studies with more samples are needed to make a better decision in this regard.
